# Nonlinear vibration of a buckled/damaged BNC nanobeam transversally impacted by a high-speed C_60_

**DOI:** 10.1038/s41598-020-80202-7

**Published:** 2021-01-12

**Authors:** Jiao Shi, Likui Yang, Jianhu Shen, Kun Cai

**Affiliations:** 1 College of Water Resources and Architectural Engineering, Northwest A&F University, Yangling, 712100 China; 2 State Key Laboratory of Structural Analysis for Industrial Equipment, Dalian University of Technology, Dalian, 116024 China; 3 School of Engineering, RMIT University, Melbourne, 3001 Australia

**Keywords:** Nanoscience and technology, Physics

## Abstract

Nanotube can be used as a mass sensor. To design a mass sensor for evaluating a high-speed nanoparticle, in this study, we investigated the impact vibration of a cantilever nanobeam being transversally collided by a high-speed C_60_ at the beam's free end with an incident velocity of *v*_In_. The capped beam contains alternately two boron nitride zones and two carbon zones on its cross section. Hence, the relaxed beam has elliptic cross section. The vibration properties were demonstrated by molecular dynamics simulation results. Beat vibration of a slim beam can be found easily. The 1st and the 2nd order natural frequencies (*f*_1_ and *f*_2_) of the beam illustrate the vibration of beam along the short and the long axes of its elliptic cross section, respectively. *f*_2_ decreases with increasing temperature. A minimal value of *v*_In_ leads to the local buckling of the beam, and a different minimal *v*_In_ leading to damage of the beam. For the same system at a specified temperature, *f*_2_ varies with *v*_In_. When the beam bends almost uniformly, *f*_2_ decreases linearly with *v*_In_. If *v*_In_ becomes higher, the beam has a cross section which buckles locally, and the buckling position varies during vibration. If *v*_In_ approaches the damage velocity, a fixed contraflexture point may appear on the beam due to its strong buckling. Above the damage velocity, *f*_2_ decreases sharply. These results have a potential application in design of a mass sensor.

## Introduction

In recent years, nanosensors have wide applications in mass measurement, gas monitor, bio-medical and chemical probes due to their excellent sensitivities^1,2^. In most nanosensors, the major components are made from carbon nanotubes (CNTs), boron nitride nanotubes (BNNTs), polysillicon, silicon nitride, or nanowires^3–6^. In mass measurement by nanosensors, the issues on assessment of the position, phase angle, and mass of a particle attract much attention. According to the geometrical boundaries, resonators for mass sensing can be classified into two types, i.e., doubly-clamped beam and cantilevered beam. For example, Li and Chou^7^ studied the two types of mass sensors from CNTs using molecular structural mechanics approach. They proposed the relationship between the natural frequency and accuracy of ~ 1 zg (1 zg = 1 × 10^–21^ g). In 2008, Chiu et al.^8^ designed a single-walled CNT-based nanosensor to measure the mass of an atom; Lassagne et al.^9^ built a clamped–clamped nanobeam from CNT, and obtained a very high accuracy frequency shift of ~ 1 Hz/yg (1 yg = 1 × 10^–24^ g) on mass responsibility, and the mass accuracy is ~ 25 zg at room temperature. Gil-Santos et al*.*^10^ tested the mass and Young’s modulus of a sample by a cantilevered silicon nanowire with ~ 1 zg of accuracy.

Considering their similar mechanical properties, e.g., extremely high in-shell modulus and strength^11,12^, to CNTs, BNNTs are also suitable for fabricating nanosensors^13–16^. Compared with CNTs, BNNTs have higher thermal stability and oxidation resistance, and its electrical properties can be adjusted by changing the contents and layout of boron and nitrogen atoms^17–20^. When replacing a part of atoms on a CNT with boron and nitrogen atoms, a boron nitride carbon nanotube (BNCNT) can be obtained^21,22^. For a BNCNT nanotube, it may not be axisymmetric, which leads to anisotropic and even chiral properties. The properties imply that a BNCNT may have better performance in mass measurement than the BNNT or CNT with the same chirality index. For example, Gil-Santos^10^ stated that both the mass and the location of an atom could be tested using a nanowire-based resonator having two orthogonal vibrations with different frequencies. When symmetrically replacing two axial zones on a CNT with boron nitride, we have a BNCNT with orthogonal stiffness in the two dimensions within its cross-section. According to Gil-Santos’s conclusion, a BNCNT-based resonator should be effective for mass sensing of a moving atom attached on it.

In those studies mentioned above, the nanoparticles stand still or move slowly on resonator. Measurement of a high-speed nanoparticle is still an open issue. Cai et al.^23^ estimated the dynamic response of a clamped–clamped BNCNT-based beam impacted by a high-speed fullerene. The minimal escaping velocity of the nanoparticle with respect to the nanobeam was found by molecular dynamics simulation approach. The minimal escaping velocity demonstrates the ability of the nanobeam for capturing a moving nanoparticle. Recently, Yang et al.^24^ discovered that the natural frequency of a BNCNT-based nanobeam decreases with the increasing of the incident velocity of nanoparticle. Before and after a bond breakage, the natural frequency of beam changed sharply. The minimal incident velocity of a nanoparticle with respect to a nanobeam was obtained after molecular dynamics simulations. This was another factor for illustrating the capability of a nanobeam in measuring a moving nanoparticle.

According to the linear beam theory, the sensitivity of a cantilevered beam is about four times higher than that of a doubly-clamped beam^4^. In this study, we adopted a cantilevered nanobeam as a resonator for evaluating a moving nanoparticle. Under the impact from a high-speed fullerene, the free end of the beam had vibration with large amplitude. The beam may buckle or be damaged after collision. The properties of the nonlinear vibration were studied by molecular dynamics simulations with consideration of the effects of the incident path of fullerene, radii of tubes, and temperature.

## Models and methodology

### Model of a BNC nanobeam

**Figure 1:**
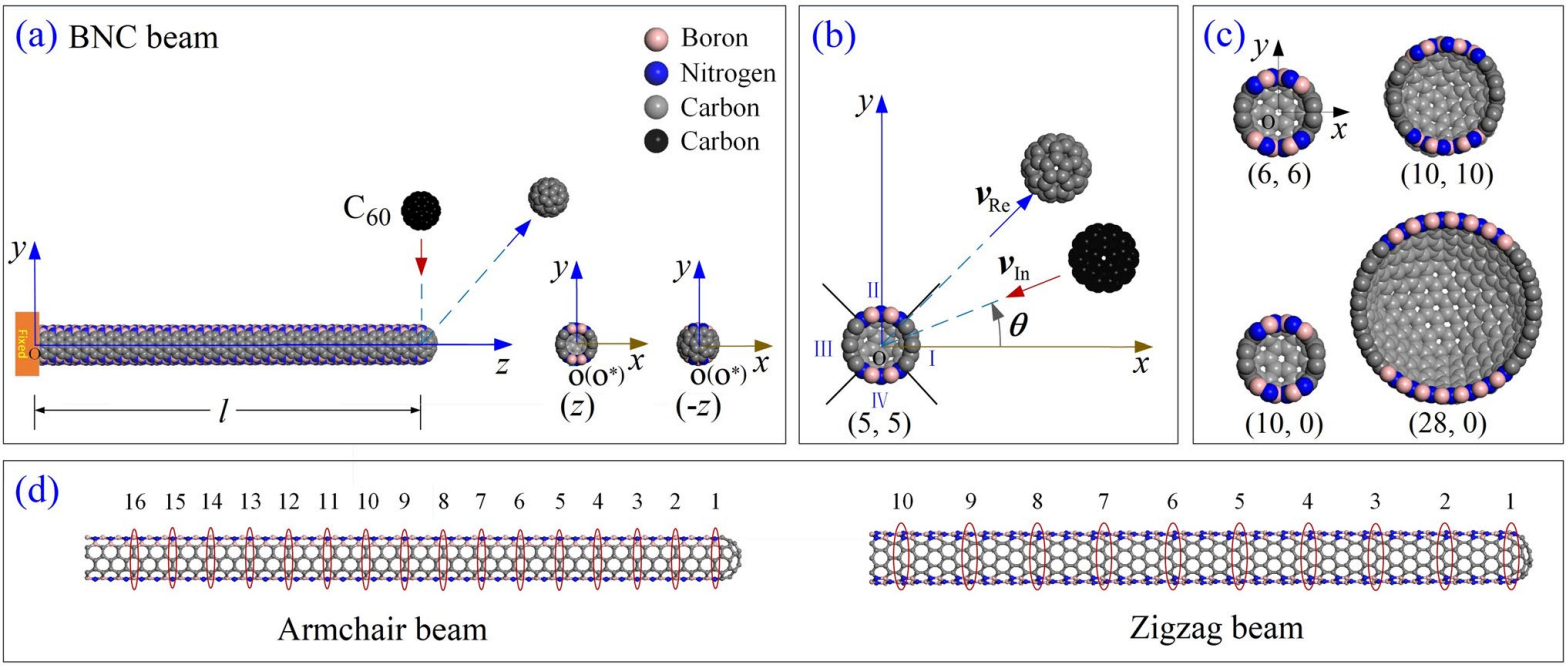
Schematic of a cantilevered beam from a capped BNC nanotube under impact of C_60_ at its free end/cap. (**a**) The beam contains two BN zones (II and IV) and two C zones (I and III). 50% of total atoms in beam are carbon atoms. C_60_ moves vertically toward the beam, and is bounced back after collision. (**b**) Incident angle *θ* is the angle between the moving path and x-axis in the xy-plane. (**c**) The caps of other four beams in tests. (**d**) Selected cross sections of the armchair and zigzag types of beams. The centroid of section 1 is labeled as “o*” with coordinates of (*x*o^*^, *y*o^*^, *z*o^*^).

The initial value of *l* is ~ 102.24 Å for the armchair beam or ~ 100.01 Å for the zigzag beam. *v*_In_ is the incident velocity of C_60_. When the fullerene moves along the reverse direction of the x-axis, i.e., *θ* = 0°, it will impact carbon atoms at zone I on the beam (Fig. 1b). If it moves along the path which is parallel to the y-axis, i.e., *θ* = 90°, it will collide with boron and nitrogen atoms at zone II. The initial distance between the mass centers of C_60_ and the cross section 1 of beam is 5 nm.

### Methodology

#### Flowchart of molecular dynamics simulation

In this study, molecular dynamics simulations were carried out using the open source code LAMMPS^25,26^ to reveal the dynamic behavior of the nanobeams as shown in Fig. 1 when its end was collided with a high-speed C_60_ fullerene. In each simulation, the following major steps were contained, i.e.,

1. Built a cantilevered BNC nanotube-based nanobeam, and a C_60_;
2. Reshaped the boundaries of the system by minimizing the potential energy of the system;
3. Fixed fullerene and six atoms on the left edge of the nanobeam, then relaxed the system at a canonical (NVT) ensemble for 200 ps;
4. After relaxation, fixed the two layers of atoms at the left edge of the nanobeam, and released C_60_ with a given velocity along a specified path. After running 0.1 ps, changed the ensemble from NVT to NVE, and recorded the dynamic responses of the beam and the fullerene within the next 30 ps. After that, changed the ensemble back to NVT and fixed C_60_. Recorded the dynamic response of the beam within the next 2000 ps;
5. Stop.

In simulation, the Tersoff potential was used to describe the interaction among the bonding atoms of carbon, boron, and nitrogen^27^. The AIREBO potential^28^ was adopted to evaluate the interaction among the carbon atoms in the system. Lennard–Jones potential^29^ was used for the estimation of non-bonding interaction between two atoms. Periodic boundary conditions were applied along the three dimensions. Timestep for integration was 0.001 ps.

#### Bi-section algorithm for finding critical values of incident velocity

For a fullerene with given incident velocity, it may be attracted and attached to the beam when the velocity is low. If a high-speed C_60_ is used, the fullerene may escape from the beam after collision^23^. However, when the incident velocity is higher than a critical value, the beam will buckle, and the minimal incident velocity which can lead to beam buckling^30,31^ is labeled as “**MIV_B**”. Note that buckling of nanotubes has been widely investigated^32–34^. If the incident velocity is much higher than MIV_B, the beam may be damaged during collision with the fullerene. Correspondingly, the minimal incident velocity of C_60_ which may lead to bond breakage in beam is labeled as “**MIV_D**”. In this study, the bi-section algorithm was applied for finding the critical value of the incident velocity in each case. Briefly, the flowchart of bi-section method contains the following steps, i.e.,

1. Give an initial interval of ***v***_In_, e.g., [*a*, *b*], where *a* and *b* satisfy Sign(*a*) × Sign(*b*) < 0;
2. Find the mean value of *c*, i.e., *c* = (*a* + *b*)/2, and calculate the value of Sign(*c*);
3. If Sign(*c*) × Sign(*a*) > 0, let *a* = *c*; otherwise, *b* = *c*;
4. If *b − a* < *η*, go to step (5); otherwise, go to step (2);
5. Stop and record *c* as the critical value of incident velocity.

where Sign(*) = 1 or − 1. For example, in calculating MIV_B, Sign(***v***_In_) = 1 means the beam buckles. If it does not buckle, Sign(***v***_In_) = *− *1. The convergence tolerance *η* is 1 Å/ps. In searching MIV_D, Sign(***v***_In_) = 1 means one or more bonds are longer than 2 Å during collision or within a period of vibration after collision. The tolerance is 0.1 Å/ps.

#### Fast Fourier transform for finding eigen frequencies

For obtaining the first few orders of frequencies of the beam after collision, the Fast Fourier Transform (FFT) approach^35–37^ was adopted on the vibration curve of the mass center of a cross section of the beam. According to the boundary condition of the beam, the cosine-expansion style was used, i.e.,

1 $$d\left( t \right) = \sum\limits_{i = 1}^{Nf} {A_{i} \cos \left( {2\pi \cdot \omega_{i} \cdot t + \phi_{i} } \right)}$$

where *d*(*t*) is the mass center of cross section 1 on the beam at time *t*. *Nf* is the maximum order of frequency in expansion. *A*_*i*_, *ω*_*i*_ and *ϕ*_*i*_ are the *i*th amplitude, frequency and phase angle, respectively.

#### Damping factor of the beam vibration

At finite temperature, the beam showed damping vibration in a NVT ensemble. During vibration, a part of structural kinetic energy was transformed into thermal vibration kinetic energy. To control the temperature to be constant, the extra thermal energy has to be removed from the system. For a resonator for mass sensing, energy dissipation influences its sensitivity^38^. To evaluate this effect, damping factor was calculated using the following formulation, i.e.,

2 $$\delta { = }\frac{1}{m}{\text{Ln}}\frac{{x_{i} }}{{x_{i + m} }},$$

where *m* is the number of periods between moments *i* and *i* + *m* with amplitudes of *x*_*i*_ and *x*_*i*+*m*_, respectively.

## Results and discussion

### Critical values of incident velocity of C_60_ at different conditions

#### Critical incident velocity of C_60_ to buckle the beam (MIV_B)

From Table 1, in a specified system with the same incident velocity and path, the value of **MIV_B** is lower than that of **MIV_D**. It means that buckling of a beam collided with C_60_ happens easier than damage. For the same system at different temperature, the value of MIV_B decreases with increasing temperature. The reason is that thermal vibration of atoms in the beam leads to weaker strength of covalent bonds in the beam. At the same temperature, the slimmer beam buckles easier. The reason is that slimmer beam is easier to be bent under the same impulse. For the same beam collided by the fullerene along different paths, the value of MIV_B with respect to *θ* = 0° is lower than that along *θ* = 90° due to greater bending stiffness of the BNCNT about the y-axis than that about the x-axis.

**Table 1:** Critical values of the incident velocity of C_60_ colliding with nanobeams at different conditions. Both the minimal incident velocities with respect to buckling beam (**MIV_B**) and with respect to damaged beam (**MIV_D**) of each case are listed.

*θ*	Chirality	Diameter	T = 8 K		T = 100 K		T = 300 K		T = 500 K	
			MIV_B	MIV_D	MIV_B	MIV_D	MIV_B	MIV_D	MIV_B	MIV_D
0°	(5, 5)	6.88	21	37.5	21	35.4	19	35.2	19	29.7
	(10, 0)	7.94	21	35.9	17	35.4	17	33.4	16	28.1
	(6, 6)	8.25	21	36.6	19	37.1	18	36.5	17	36.8
	(10, 10)	13.75	21	52.1	18	51.0	16	50.0	15	49.8
	(28, 0)	22.23	34	96.6	33	93.1	31	92.9	29	83.9
90°	(5, 5)	6.88	33	44.6	31	43.7	31	43.1	26	35.0
	(10, 0)	7.94	34	54.6	33	55.1	32	50.8	31	49.3
	(6, 6)	8.25	30	55.6	30	46.3	28	45.0	28	43.8
	(10, 10)	13.75	28	82.4	27	81.7	24	78.3	23	69.9
	(28, 0)	22.23	34	95.1	33	94.0	33	84.7	32	92.0

Unit of dimension: Å. Unit of velocity: Å/ps.

#### Critical incident velocity of C_60_ with respect to damaged beam (MIV_D)

Using bisection algorithm, the critical values of incident velocity of C_60_, which can damage the nanobeam, were obtained after MD simulations. The results shown in Table 1 indicate that the value of MIV_D decreases with increasing temperature when a BNC beam is impacted by a C_60_ along the same path. For example, at 8 K, a defect is generated on the (5,5) nanobeam when subjected to collision of C_60_ with velocity of 37.5 Å/ps, which is higher than that at 500 K. The reason is that at higher temperature, the bond strength is weakened by stronger thermal vibration of atoms. In general, the bond is considered as being broken when its length is larger than 2 Å, and is never regenerated again. Hence, at higher temperature, the C_60_ with lower incident velocity can induce bond breakage in beam. At the same temperature, the nanobeam with smaller radius, in general, will be damaged easier, i.e., the value of MIV_D is lower. For instance, at 300 K, the (5,5) nanobeam is damaged by the C_60_ with velocity of 35.2 Å/ps, which is lower than 50 Å/ps with respect to the (10,10) nanobeam impacted by the same fullerene along the same path. The reason is that when *v*_In_ and *θ* keep constant, a slimmer beam has larger curvature, which means that some bonds in beam are under heavier tension along the circumferential direction. Hence, the C_60_ with lower incident velocity can damage the slimmer beam at the same temperature.

When comparing the effect of incident path, one can find that the same beam is damaged by the C_60_ with higher incident velocity moving along *θ* = 0° than along *θ* = 90°. It is because the fullerene impacted at the zone I ( Fig. 1b) where C–C bond is stronger than that at zone II with B–N bonds with respect to the path of *θ* = 90°. On the other hand, chirality of nanobeam also influences the value of MIV_D when C_60_ moves along *θ* = 90° and impacts the two beams with slight difference of their radii. For example, at 300 K, (10,0) nanobeam behaves stronger than (5,5) or (6,6) nanobeam according to the results in Table 1.

During searching the values of MIV_D as listed in Table 1, we also found some special characteristics of the beam’s vibration during and after collision with the high-speed C_60_. The vibrations of beam with or without defect are different. To demonstrate these characteristics, both undamaged and damaged beams were discussed below.

### Vibration of a slim beam when MIV_B < ***v***_In_ < MIV_D

According to the results listed in Table 1, the values of MIV_B and MIV_D are 33 Å/ps and 55.1 Å/ps, respectively, for the system with the (10,0) beam with *θ* = 90° at 100 K. Here, two values of *v*_In_, e.g., 35 Å/ps and 55 Å/ps were considered in discussion.

**Figure 2:**
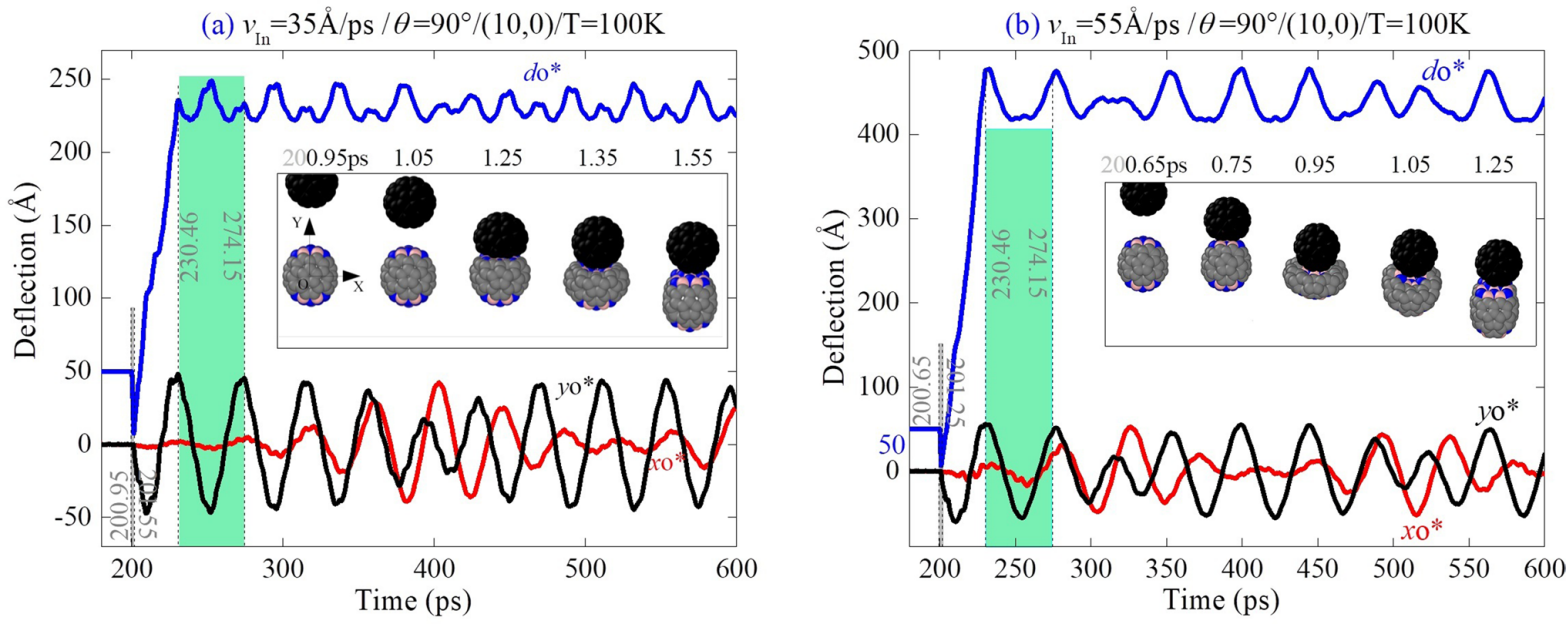
Vibration of the (10,0) beam tip (cross section 1) and motion of C_60_ before and after collision with different incident velocities of (**a**) *v*_In_ = 35 Å/ps, and (**b**) 55 Å/ps when *θ* = 90° at 100 K. o^*^ is the centroid of cross section 1 in the beam. “*d*o^*^” (blue curves) is the distance between the centroid of C_60_ and o^*^ with coordinates of *x*o* (red curves), and *y*o* (black curves) in the vibration planes. Insets are snapshots during collision.

When the beam is impacted by the C_60_ along the path of *θ* = 90°, the boron and nitrogen atoms on the beam section 1 are collided with the fullerene. According to the insets in Fig. 2, both the fullerene and the free end of the beam experience four steps, i.e., contacting, deforming, moving synchronously, and bouncing back. The insets also demonstrate that the collision between the two components belong to central impact, i.e., the line through their centroids is in the yz-plane and parallel to the y-axis.

From Fig. 2, one can find that the fullerene stops moving at 230.46 ps, i.e., ~ 30 ps after collision. From then on, the beam starts self-vibrating. The first period of self-vibration is between 230.46 and 274.15 ps. Figure 2a implies that when *v*_In_ = 35 Å/ps, the maximum deflection of beam section 1 is ~ 50 Å. Simultaneously, another transverse vibration in the xz plane is generated, and the amplitude of vibration (xo*) is close to that in the yz plane (yo*) after about three periods of vibration. Later, both amplitudes fluctuate as time goes. It demonstrates that the beam has a self-excited beat mode after central impact at zone II, i.e., a BN zone. The trace of the beam tip has a whirling motion during vibration (Movie 1).

**Figure 3:**
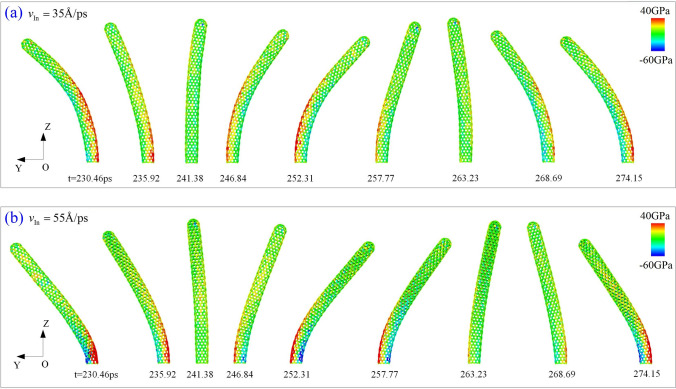
Snapshots in the first period of vibration of the nanobeam after colliding with C_60_. When incident velocity is (**a**) 35 Å/ps, and (**b**) 55 Å/ps.

When *v*_In_ = 55 Å/ps ( Fig. 2b), the beam deforms greater and the amplitude in the yz-plane is larger than 60 Å. According to the modes and the distributions of the axial (virial) stress in the beams shown in Fig. 3, the modes of beam are different slightly, but the stress distribution near the fixed end of the beam is different obviously. It is because the beam has larger bending deformation when the incident velocity is higher. Results in Table 1 have confirmed that the beam will break once *v*_In_ > MIV_D. Note that the forepart of the beam keeps straight during vibration when *v*_In_ = 55 Å/ps. This is different from that in the beam when *v*_In_ = 35 Å/ps, i.e., the beam bends almost uniformly.

**Figure 4:**
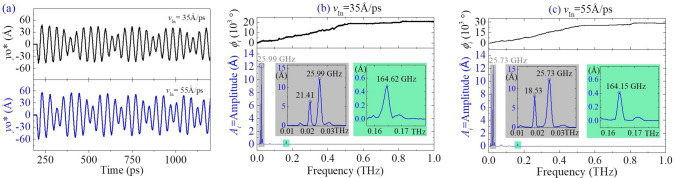
FFT results of yo*(t) curves with respect to different incident velocities. (**a**) Vibrations of the beam in the yz-plane when *v*_In_ = 35Å/ps and 55Å/ps, respectively. (**b**,**c**) Results of FFT in the time interval of [200, 2200] ps.

In Fig. 2b, beat phenomenon is also observed directly. To illustrate the beat modes observed above, the FFT results are given in Fig. 4. The beam has the first order frequencies of 21.41 GHz and 18.53 GHz at the two different incident velocities, respectively. The second order frequencies are 25.99 GHz and 25.73 GHz, respectively. The third order frequencies are 164.62 GHz and 164.15 GHz, respectively. According to our previous study^24^, the vibration of a beam section can be considered as combination of three different vibrations, i.e., the structural vibration of beam at gigahertz level, the 100 GHz level of breathing vibration of cross section and the terahertz thermal vibration of atoms on the section. Hence, both the first and the second orders of frequencies belong to transversal vibration of the structure. The third order frequencies reflect breathing vibration of the cross section 1. Thermal vibration of atoms has extremely small amplitude as compared with those of the first three modes. Hence, the related frequencies are not given in FFT processes.

For the nanobeam from BNC nanotube, it contains two BN zones and two C zones. The two types of zones have different mechanical behavior. According to our previous study^23^, the beam has an elliptic cross-section with the short and long axes along x- and y-axes, respectively. Hence, moment of inertia about the y-axis, i.e., *I*_y_, is lower than that about the x-axis, i.e., *I*_x_. Meanwhile, the natural frequency (*f*) of a beam is proportional to $\sqrt {EI/(\rho AL^{4} )}$, where *E* is the Young’s modulus, *I* the moment of inertia, $\rho$ the mass density, *A* the area of cross-section and *L* the span of the beam, respectively. Hence, *f*_y_/*f*_x_ = $\sqrt {I_{{\text{y}}} /I_{{\text{x}}} }$ > 1.0, where *f*_y_ and *f*_x_ are the natural frequencies of vibration about the y- and x-axes, respectively. Hence, the first order of frequency reflects the vibration of beam in the xz-plane, and the second order frequency describes the vibration in the yz-plane. One can find that the eigen frequencies decrease with the increasing *v*_In_.

In particular, the first order frequencies are different greatly. It is due to the beam with respect to *v*_In_ = 55Å/ps has larger deformation after collision. But the second order frequencies are different slightly because the vibration amplitude of the beam in the xz-plane is relatively lower. The differences illustrate a fact that the natural frequency of the beam reduces with increasing deflection.

### Frequency spectrum of the vibration of a slim beam

#### Zigzag beam

**Figure 5:**
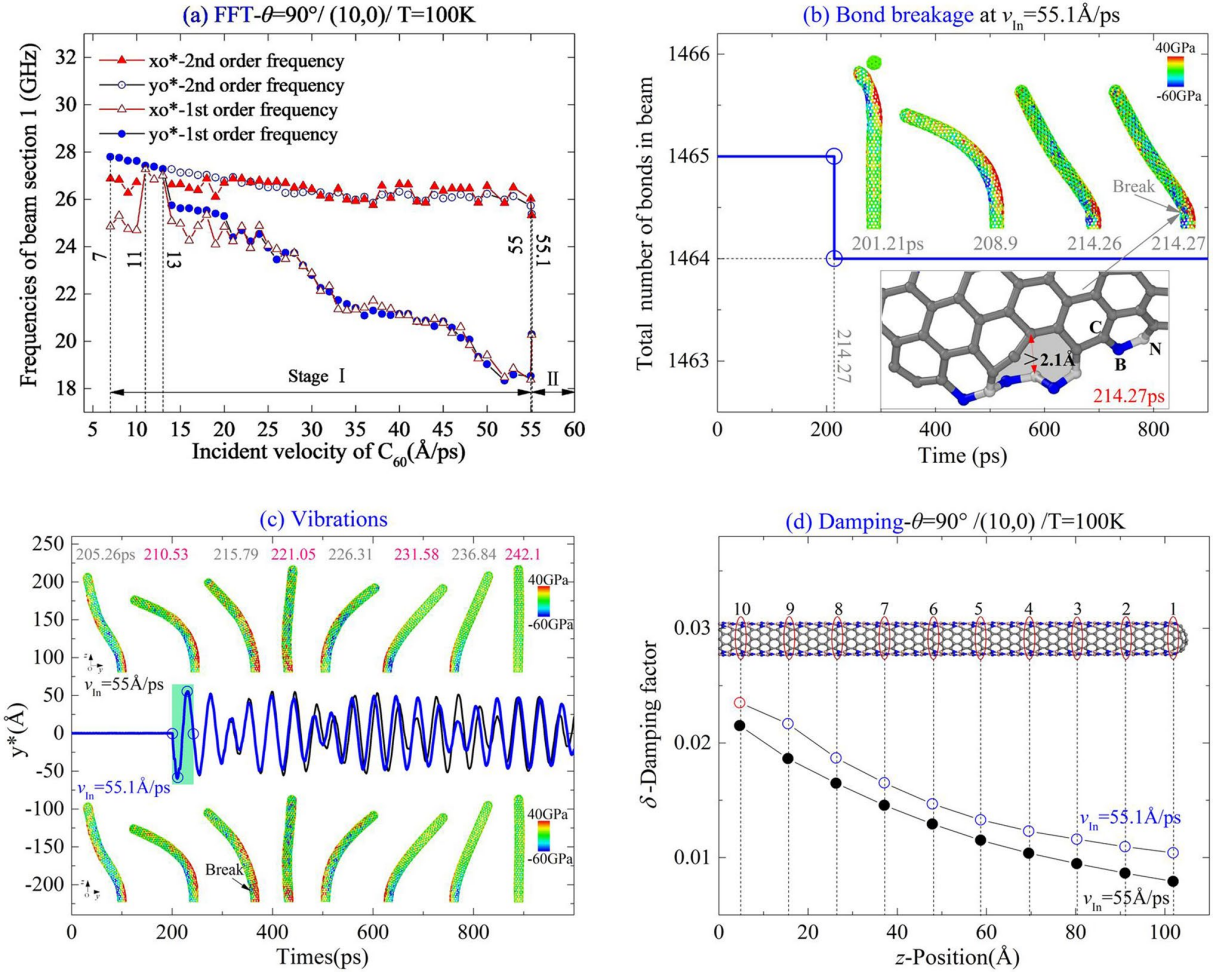
Vibration analysis of (10,0) nanobeam impacted by a high-speed C_60_ along the path of *θ* = 90° at 100 K. (**a**) Natural frequencies of the beam impacted by the C_60_ with different incident velocities. (**b**) At critical incident velocity, i.e., 55.1 Å/ps, the beam has a breakage of bond C–N near its fixed end. (**c**) Comparison of beam modes with respect to the incident velocities nearby the critical value. (**d**) Damping factors of beam sections.

Here, the same system as mentioned above is involved in discussion. For the beam with CNT (10,0) collided with C_60_ along the path of *θ* = 90°, when *v*_In_ = MIV_D, i.e., 55.1 Å/ps at 100 K, the breakage of B–C bond happens between section 9 and section 10 ( Fig. 5b).

By FFT on both xo*(*t*) and yo*(*t*), the natural frequencies of beam section 1 are obtained with respect to different incident velocities between 7 and 55.1 Å/ps. According to the results in Fig. 5a, the difference between the results of FFT on yo*(*t*) and xo*(*t*) are slight, hence, the FFT results are reliable. When incident velocity is less than 20 Å/ps, the second order frequency is sensitive to the incident velocity. After that, the second order frequency depends slightly on the incident velocity of fullerene when the beam is not damaged after collision. Note that between 11 and 13 Å/ps, the second order frequency disappears. For the first order frequency of the beam, it starts to decrease almost linearly with increasing incident velocity that is higher than 13 Å/ps. It demonstrates that the beam becomes softer when the C_60_ introduces greater kinetic energy. The reason is that the beam deformed larger (see the snapshot at 205.26 ps in Fig. 5c), and the beam is in nonlinear vibration with large-rotation of its cross-section, which is not considered in linear vibration.

If *v*_In_ > 55 Å/ps, e.g., 55.1 Å/ps, a C–N bond is broken at 214.27 ps ( Fig. 5b,c), and the location is between Sects. 9 and 10. The breakage of bond is caused by three reasons. First, after collision, the beam has large curvature which leads to heavy tensile stress on a part of covalent bonds. If the stress-induced elongation of a bond is higher than 2 Å, the bond breaks. Second, both C–N (bonding energy 2.83 eV) and C–B (2.59 eV) are weaker than C–C (3.7 eV) and B–N (4 eV)^39^. At the interface between the BN zones and C zones, C–B and C–N are under complicated stress state due to beat vibration of beam. Final, thermal vibration of the atoms in the beam leads to random distribution of thermal strain on the beam. By superposition of both bending deformation and thermal vibration, a C–N or C–B will break. However, the location of the broken bond is not determined by the incident velocity when considering the random effect of thermal vibration.

Impacted by the C_60_ with respect to different incident velocities, the beam has a slower vibration in the yz-plane, but faster in the xz-plane once the beam is damaged (Fig. 5a,c). The reason is that the broken bond results in rotation of the principal axes from the original x-/y-axes. Besides, the bonds nearby the broken bond are relaxed after bond breakage, and this reduces the local stiffness. Meanwhile, the bending stiffness about the new short-axis x’ increases, but the stiffness about the long axis y′ reduces. Hence, the ratios of moment of inertia satisfy *I*_x′_/*I*_y′_ < *I*_x_/*I*_y_, i.e., *f*_y′_/*f*_x′_ < *f*_y_/*f*_x_.

In Fig. 5d, the damping factor of the undamaged beam is smaller than that of the damaged beam, and the two factors are very low. Meanwhile, the damping factor of a beam section is lower when it is further away from the fixed end.

#### Armchair beam

For the beam from CNT (5,5) collided with C_60_ with *θ* = 0°, it buckles at *v*_In_ = 21 Å/ps, and is damaged at 37.5 Å/ps at 8 K according to the result listed in Table 1. Breakage of bond C–C happens in zone I and between section 16 and the fixed end.

**Figure 6:**
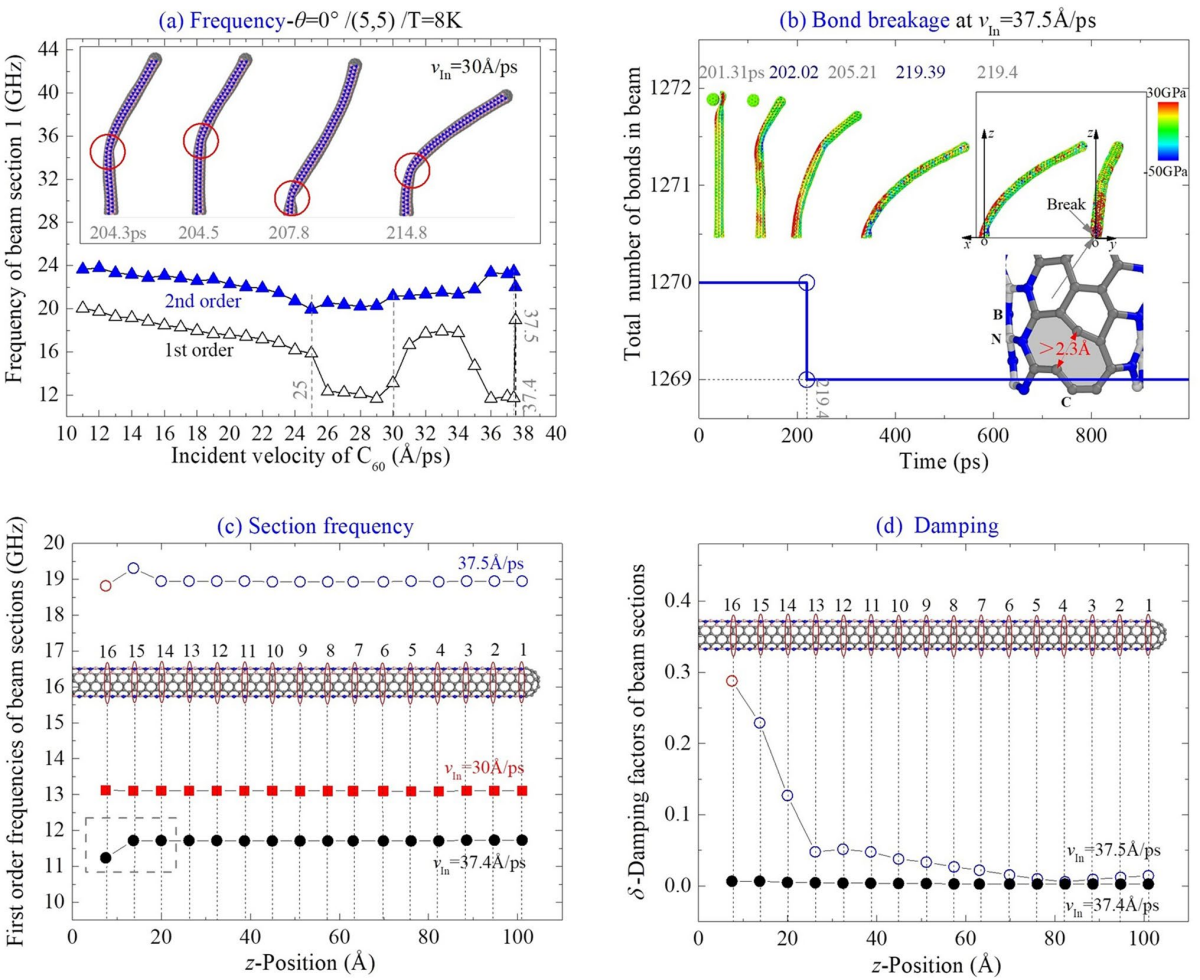
Vibration analysis of (5, 5) the nanobeam impacted by a high-speed C_60_ along the path of *θ* = 0° at 8 K. (**a**) Natural frequencies of the beam impacted by the C_60_ with different incident velocities. Snapshots of buckled beam are inserted. (**b**) At MIV_D = 37.5 Å/ps, beam has a breakage of bond C–C in the zone I near the fixed end. (**c**) Comparison of frequencies of beam sections with respect to different incident velocities. (**d**) Damping factors of beam sections.

In Fig. 6a, both the first and the second order frequencies of the beam section 1 decrease till the beam buckles at *v*_In_ = 21 Å/ps < 25 Å/ps, with linear decreasing of the first order frequency of the beam. When MIV_D > *v*_In_ > 25 Å/ps, that the second order frequency increases till the beam has bond breakage nearby its fixed end, while, the magnitude of first order frequency fluctuates. Before buckling, the vibration amplitude of the beam increases with incident velocity. However, if the beam buckles, the local buckling shortens the vibrating span in the xoz plane. Hence, the first two order frequencies increase when *v*_In_ ≤ 34 Å/ps. When *v*_In_ > 34 Å/ps, the beam deflection in the xz-plane is too large, hence, the frequency decreases with increasing *v*_In_.

When a C–C bond breaks in zone I nearby the fixed end, the shape of section 16 varies, and the moment of inertia about the y-axis decreases, or increases about the x-axis. Hence, the first order frequency increases but the second decreases. Here, we demonstrated an interesting phenomenon, i.e., the buckling location of the beam varies in vibration (Movie 2). This is because a slimmer beam buckles easier when the forepart of beam attracts higher kinetic energy.

In Fig. 6b, the location of the bond breakage is illustrated, i.e., at 219.4 ps, a C–C bond in zone I nearby the fixed end breaks when *v*_In_ = 37.5 Å/ps. When the beam has bending deformation shown as shown in the snapshot, zone I is under the largest tension according to beam theory, hence, the bonds in this region break easier. At extremely low temperature, i.e., 8 K, the influence of thermal vibration can be neglected. Hence, the neighbor C–B or C–N bonds do not break. By comparing the section’s first order frequencies at different conditions (Fig. 6c), we concluded that variation of the local bending stiffness caused by either buckling or bond breakage leads to the differences among the frequencies of the beam sections. For example, when *v*_In_ = 37.4 Å/ps, the frequencies of the sections closer to the broken bond are different from those of remained sections whose frequencies are identical. And the damping factor ( Fig. 6d) varies sharply at the cross section with reduced bending stiffness.

**Figure 7:**
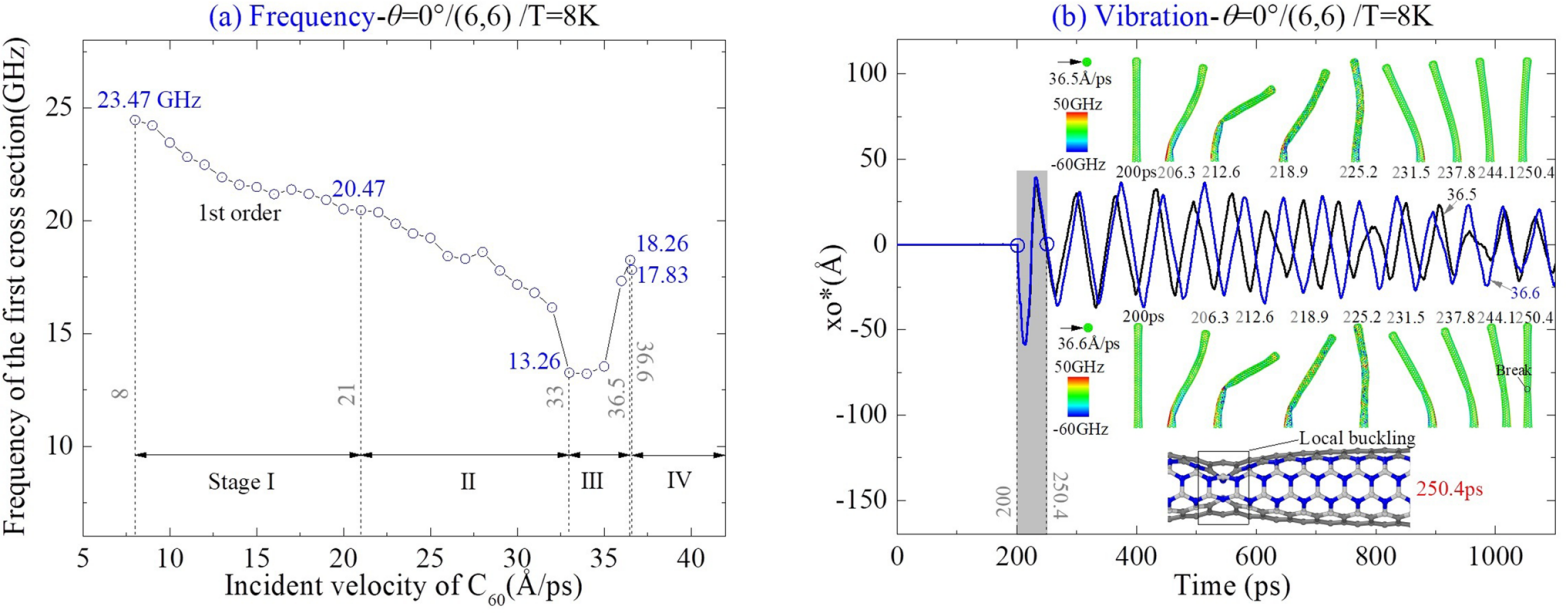
Vibration analysis of (6,6) the nanobeam impacted by C_60_ along the path of *θ* = 0° at 8 K. (**a**) Natural frequency of the beam versus the incident velocity of C_60_. (**b**) Vibration curves of cross section 1 when the incident velocity is nearby MIV_D = 36.6 Å/ps, Breakage of bond appears between cross Sects. 11 and 12. Snapshots of beam are inserted.

For comparison, the beam from CNT (6,6) collided with C_60_ along the same path, i.e., *θ* = 0°, is discussed on its dynamic response. According to the results in Fig. 7a, the beam has bond breakage when *v*_In_ = 36.6 Å/ps at 8 K, i.e., MIV_D = 36.6 Å/ps. Meanwhile, the local buckling on the beam happens when the incident velocity is 21 Å/ps (MIV_B). When the value of *v*_In_ is close to MIV_D, the beam has a contraflexture point in the middle part of it due to seriously local deformation (see the snapshot at 212.6 ps when *v*_In_ = 36.5 Å/ps in Fig. 7b). This is significantly different from the buckling configuration of the beam with CNT (5,5) by comparison with the snapshots in Fig. 6b. The reason is that the radius of CNT (6,6) is larger than that of CNT (5,5). During deformation of CNT (6,6), the repulsion of the internal surface at the contraflexture point does not increase obviously after generation of the local puckering. Besides, this damage is unrecoverable.

### Frequency spectrum of the vibration of a thick beam

#### Armchair beam impacted along different paths

##### Along the path of *θ* = 90°

For the beam from CNT (10,10) collided with C_60_ along *θ* = 90° at 300 K, it buckles before bond breakage appears between beam Sects. 14 and 15 (close to the fixed end) when MIV_D = 78.3 Å/ps ( Table 1).

**Figure 8:**
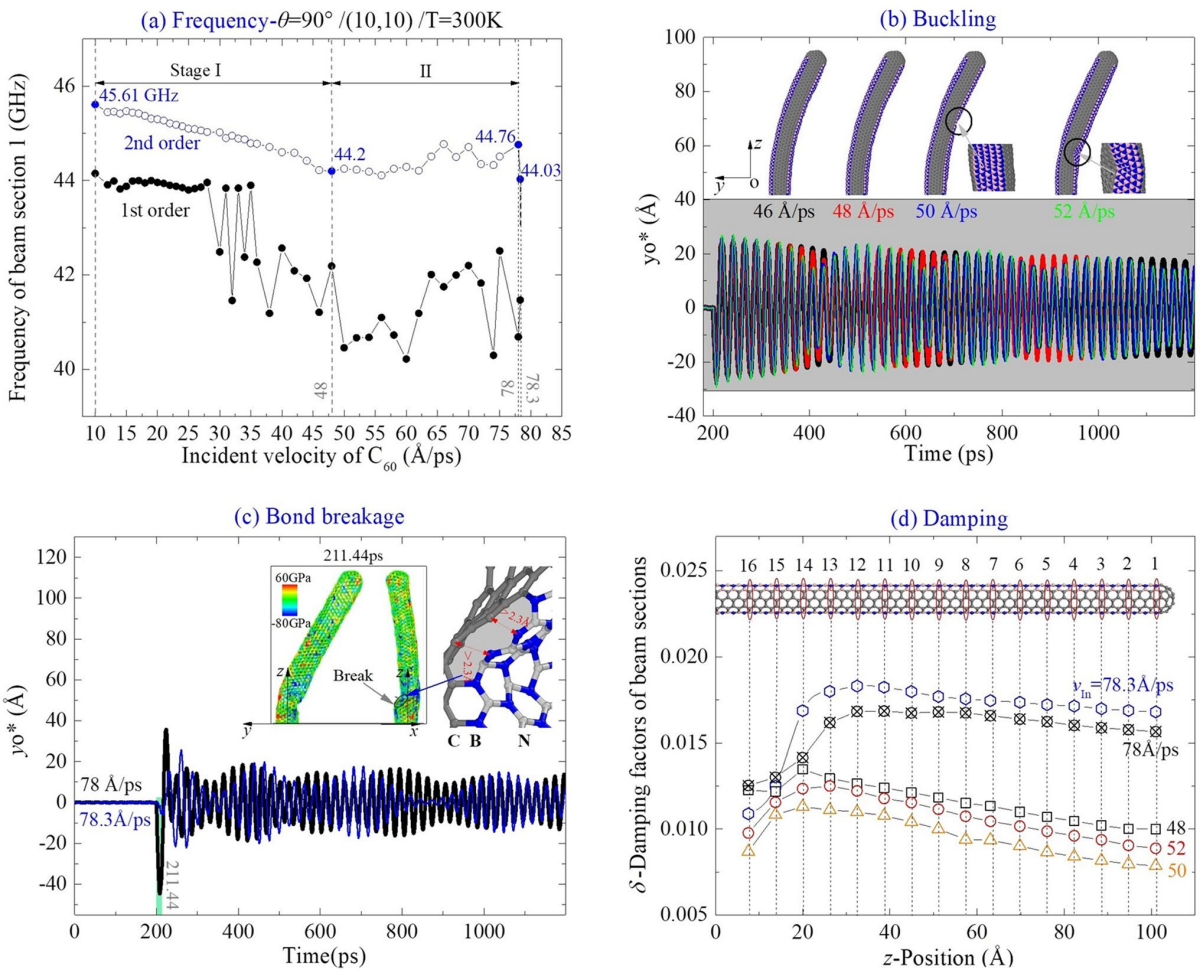
Vibration analysis of (10,10) beam impacted by a C_60_ along the path of *θ* = 90° at 300 K. (**a**) Natural frequencies of the beam impacted by the C_60_ with different incident velocities. (**b**) Vibration of the beam nearby MIV_B = 24 Å/ps. (**c**) At MIV_D = 78.3 Å/ps, two C–B bonds break between beam Sects. 14 and 15. (**d**) Damping factor of beam sections with respect to different incident velocities.

In Fig. 8a, the first two order frequencies are different, which indicates that the beam is in beat vibration (see the vibration curves in Fig. 8b). The second order frequency decreases with the incident velocity between 10 Å/ps (< MIV_B) and 48 Å/ps (> MIV_B). However, when the beam buckles seriously, e.g., when *v*_In_ = 50 Å/ps (see the inset in Fig. 8b) it increases with the incident velocity.

According to the inserts in Fig. 8b and Table 1, the beam has obvious local buckling when *v*_In_ is slightly higher than 50 Å/ps. At the local buckled area, the beam has larger deformation when the incident velocity is higher. If the beam is impacted by the C_60_ with *v*_In_ ≥ 78 Å/ps, two C–B bonds break between beam Sects. 14 and 15 on the boundary of zones I and II (Fig. 8c). The initially maximal deflection of beam in the y–z plane becomes much higher. This is because the local bond breakage leads to larger rotational angle of the free end of the beam after collision. From the snapshot in the xz-view, one can also understand that the beam has deflection in the x-direction, which is also an evidence of beat phenomenon.

In Fig. 8d, the damping factor of the beam sections varies faster nearby the damaged area, i.e., between Sects. 14 and 15. Once the beam section is far from the damaged area, its damping factor decreases slowly. This implies a method for measuring the location of the damaged area.

##### Along the path of *θ* = 0°

**Figure 9:**
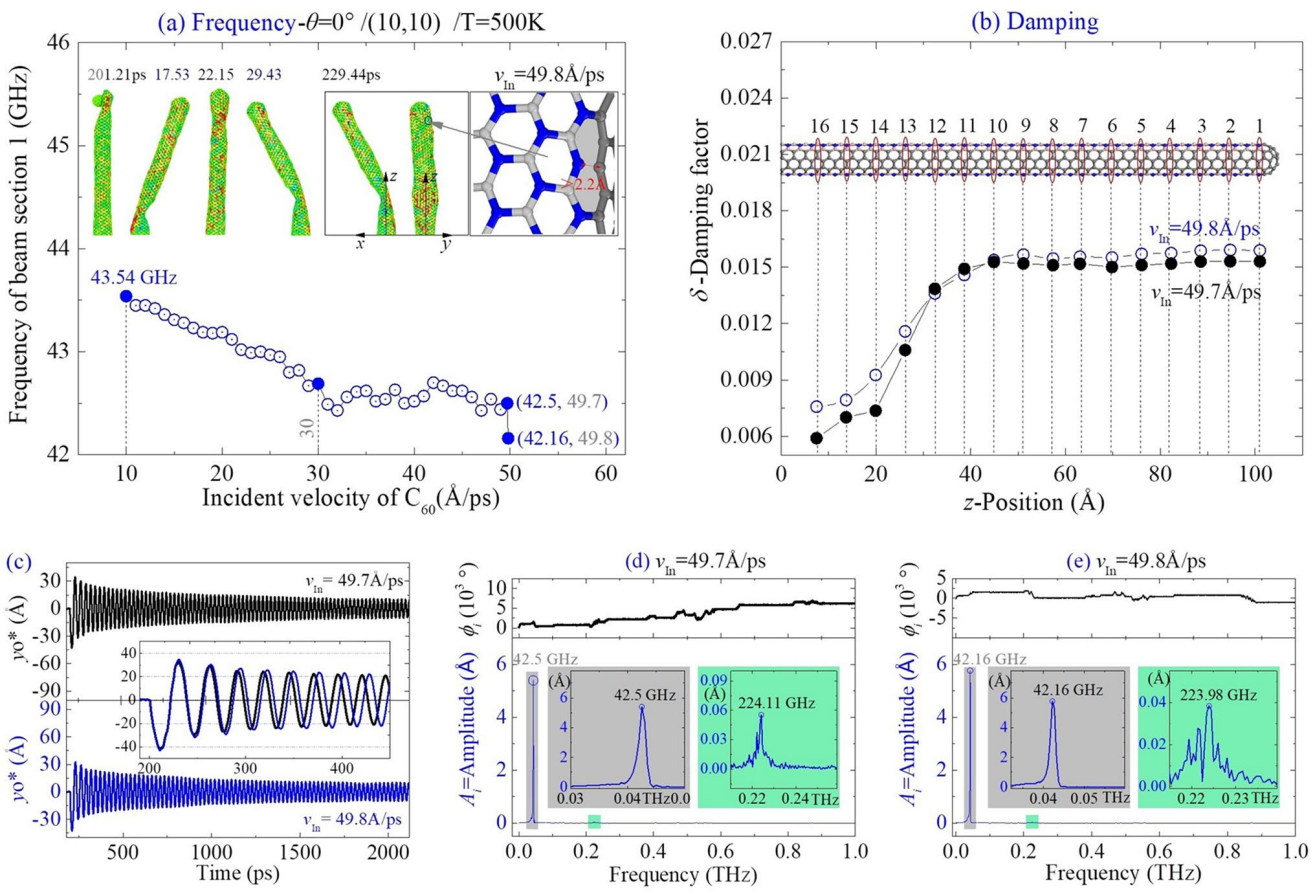
Vibration analysis of (10,10) nanobeam impacted by a high-speed C60 along the path of *θ* = 0° at 500 K. (**a**) Natural frequencies of the beam impacted by the C60 with different incident velocities. (**b**) Damping factors of beam sections. (**c**) Vibration curves of yo*, and (**d**,**e**) the FFT results.

For the beam with CNT (10,10) collided by C_60_ along *θ* = 0° at 500 K, it buckles slightly at *v*_In_ = 15 Å/ps (MIV_B) and heavily at 30 Å/ps. When the incident velocity reaches 49.8 Å/ps (MIV_D), bond breakage of C–B happens between section 2 and section 3 at the boundary of zones III and IV (Fig. 9a). As the damaged area is near to the beam top, it slightly influences the damping factor of a section ( Fig. 9b). If the beam is not damaged, its first order frequency is between 42.43 and 42.7 GHz, i.e., the incident velocity has no influence on the eigen frequencies of beam.

According to the FFT results shown in Fig. 9d,e, the vibration of beam with respect to *v*_In_ = 49.7 or 49.8 Å/ps has no beat phenomenon( Fig. 9c). It demonstrates that a thick beam had no beat vibration when it is impacted at zone I or III. And the damaged area located at the beam top also has slight influence on the magnitude of the first order frequency.

#### Zigzag beam

Here, CNT (28,0) is chosen to form a thick beam. Meanwhile, the incident path of C_60_ is along *θ* = 90°. The dynamics response of the beam at 300 K was investigated. According to the results shown in Table 1, MIV_B = 33 Å/ps, and MIV_D = 84.7 Å/ps for the beam. The value of MIV_B with respect to *θ* = 90° is slightly higher than that with respect to *θ* = 0°. However, the value of MIV_D is much higher than that with respect to *θ* = 0°. It is caused by different bending stiffness of the beam along the x- and y-axes. Briefly, the beam contains two C-zones and two BN-zones, and the cross section of the beam becomes an ellipsoid with long axis along the y-axis. Hence, the beam has the same large displacement on its tip in the y–z plane on condition that the C_60_ introduces greater kinetic energy.

**Figure 10:**
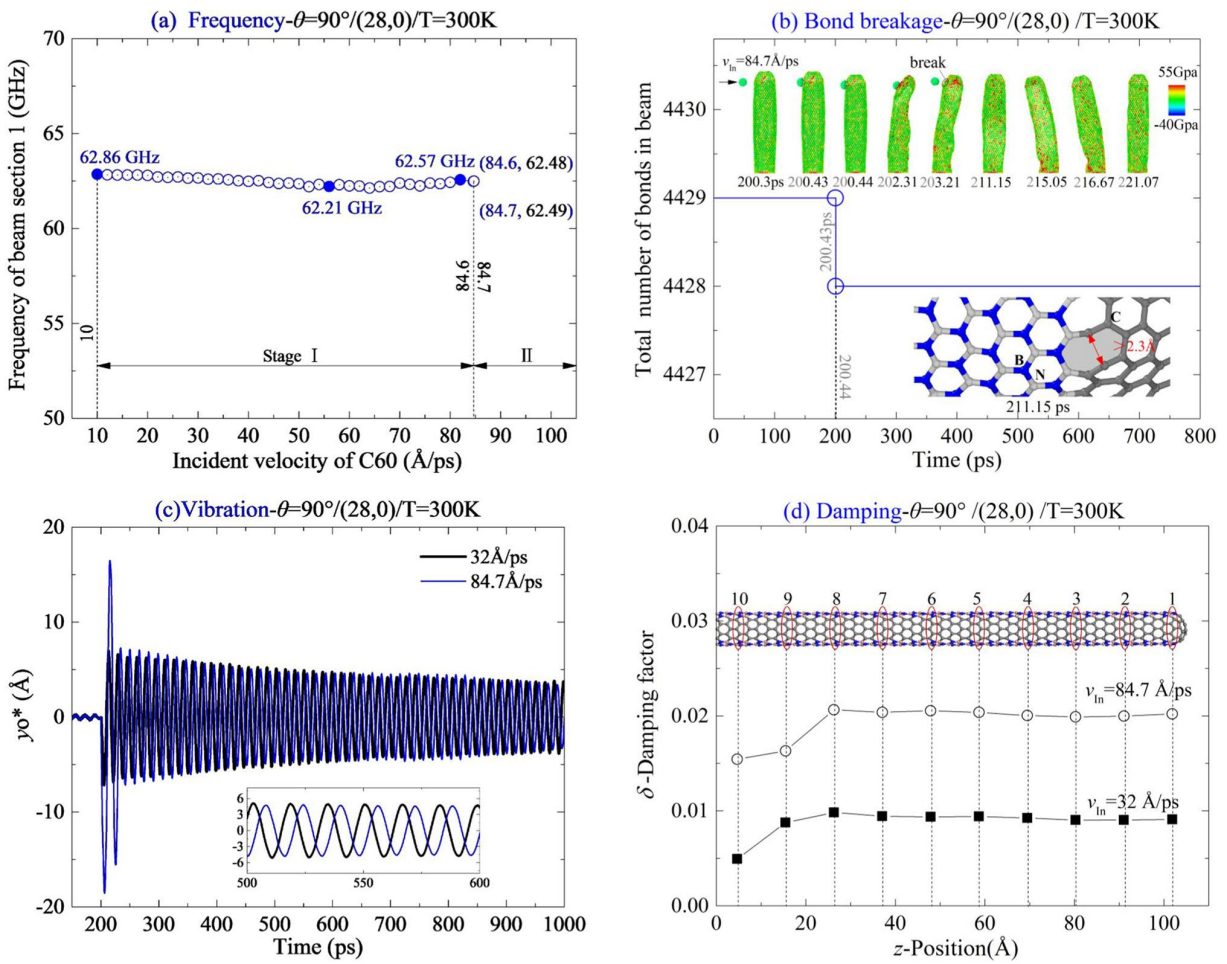
Vibration analysis of (28,0) nanobeam impacted by a high-speed C60 along the path of *θ* = 90° at 300 K. (**a**) Natural frequencies of the beam impacted by the C60 with different incident velocities. (**b**) Damping factors of beam sections. (**c**) Vibration curves of yo*,when *v*_In_ = 32 Å/ps and 84.7 Å/ps. (**d**) Damping factors of beam sections.

By inspection of the vibration process of the beam after collision with the fullerene with different incident velocities, e.g., 32 Å/ps and 84.7 Å/ps, the vibration of beam changes slightly (Fig. 10a,c). The reason is that, the bond breakage appears near the free end of the beam (see the snapshot at 203.21 ps in Fig. 10b). The damping factor of the two velocities are obviously different (Fig. 10d). As the tube has larger radius, deformation of the thick shell becomes more complicated than that on a slim tube, e.g., CNT (10,0) in Fig. 5c. Especially, the vibration of beam has no beat phenomenon, which appears in a slim zigzag beam.

## Conclusions

Nanotubes have been successfully applied as a mass sensor for measuring a molecular for years. But using a nanotube-based mass sensor to evaluate the mass of a high-speed nanoparticle is still an open issue. In this study, we investigated the vibration of a cantilever nanobeam from a capped boron nitride carbon nanotube (BNCNT) being impacted by a high-speed C60 at the free end. By molecular dynamics simulations, we found some interesting dynamic characteristics of the beam during vibration after collision. The following conclusions will be potentially applied in design of such mass sensor, i.e.,

1. The minimal incident velocity of C60 is called MIV_B when the beam has local buckling after collided by the fullerene. If the incident velocity is higher than MIV_B, the beam may be damaged by the fullerene after collision. The corresponding minimal value is called MIV_D. For the same system at different temperature, the values of MIV_B and MIV_D decrease with increasing temperature. At the same temperature, the slimmer beam buckles easier.
2. The cross-section of the relaxed beam is elliptic, and beat phenomenon can be found in the vibration of a slim beam or a beam impacted at it BN zone (*θ* = 90°). The first and the second order frequencies describe the vibrations of the beam along the short axis and the long axis, respectively. For the same system, e.g., the same beam, the same incident path, and the same temperature, the first order frequency fluctuates severely when the incident velocity changes between MIV_B and MIV_D. The second order frequency of the beam decreases firstly in linear with the incident velocity, and then fluctuates, and finally jumps down sharply. Linear decreasing of the natural frequency is caused by large deformation of the beam with weakly local buckling. Fluctuation of the natural frequency is caused by serious buckling of the beam after collision, while, down-jumping is caused by bond breakage in the beam.
3. If the beam bends almost uniformly during vibration, the frequency decreases linearly with the incident velocity. When the incident velocity is higher, the position of buckling on the cross section may vary during vibration. If the incident velocity is close to MIV_D, the buckling position does not change on the beam during vibration.
4. On the same beam, the frequency of the buckled or damaged cross section is lower than that of the undamaged sections. Simultaneously, the damping factor of the buckled or damaged section will be different from that with an undamaged section.

## Supplementary Information

Supplementary Movie 1.

Supplementary Movie 2.

## Data Availability

The raw/processed data required to reproduce these findings cannot be shared at this time due to legal reasons.
